# Editorial: Strengths and Challenges of Allo-SCT in the Modern Era

**DOI:** 10.3389/fonc.2022.850403

**Published:** 2022-02-24

**Authors:** Michele Malagola, Raffaella Greco, Jacopo Peccatori, Alessandro Isidori, Rizwan Romee, Mohamad Mohty, Fabio Ciceri, Domenico Russo

**Affiliations:** ^1^Unit of Blood Diseases and Stem Cell Transplantation, ASST-Spedali Civili di Brescia, Department of Clinical and Experimental Sciences, University of Brescia, Brescia, Italy; ^2^Unit of Hematology and Bone Marrow Transplantation, IRCCS San Raffaele Scientific Institute, Vita-Salute San Raffaele University, Milan, Italy; ^3^Hematology and Stem Cell Transplant Center, AORMN Hospital, Pesaro, Italy; ^4^Division of Hematologic Malignancies, Dana-Farber Cancer Institute, Boston, MA, United States; ^5^Department of Hematology and Cellular Therapy, “Saint Antoine Hospital” AP-HP, Paris, France

**Keywords:** allogeneic SCT, relapse prevention, immune reconstitution, cytomegalovirus (CMV), CART

The use of allogeneic stem cell transplantation (allo-SCT) is increasing over time worldwide (1, 2) and this is the result of three major facts: i) the evidence that allo-SCT can cure a progressively higher number of patients and many hematological diseases (1, 2); ii) the increase in the upper limit of age for allo-SCT eligibility (currently up to 75 years) (3); iii) the improvement of allo-SCT platforms both in terms of efficacy and toxicity (4, 5). However, many challenges in the field of allo-SCT still need to be overcome, mainly the reduction of relapse rate and of transplant related-mortality. Currently, we can assume that approximately 50% of the patients can be cured by allo-SCT, taking into account all the favorable and unfavorable variables associated with long-term outcome after transplantation (6).

The aim of this Research Topic entitled Advances and Challenges of allo-SCT was to collect articles that highlights the most recent evolutions of the transplant platform, together with a focus on the most intriguing new insights that will be developed in the next future. Twenty-one manuscripts have been submitted and eighteen out of them have been accepted for publication.

Interestingly four of these manuscripts cover the field of relapse prevention and treatment, either with donor-lymphocyte infusion (DLI) or with de-methylating agents. Su et al. reports on two strategies on prophylactic DLI in patients with relapsed-refractory acute leukemias: infusion from day +60 irrespective of minimal residual disease (MRD) (cohort 1) and on day +60 or +90 basing on MRD (cohort 2). In summary, they showed that a delay of prophylactic DLI up to day +90 basing on MRD could be associated with lower extensive cGVHD and better graft and relapse-free survival (GRFS). In the survey by the Gruppo Italiano Trapianto di Midollo Osseo (GITMO) published by Patriarca et al. on 254 patients with acute leukemias, 73% of the cases received DLI for leukemia relapse and only 10% received DLI as pre-emptive treatment. Nevertheless, by multivariate analysis, a pre-emptive use of DLI without evident leukemia relapse and multiple infusions of lymphocytes were associated with improved overall survival. In the last 15 years, several papers have been published on the topic of DLI. However, results have been contradictory, and the risk of graft versus host disease (GVHD) following lymphocytes infusion has hampered the extensive and homogeneous use of DLI as post-transplant adoptive immunotherapy. As a consequence, DLIs have been generally used to cure overt hematological relapse. Nevertheless, the use of DLI driven by MRD assessment seems to be the challenge for the next future (7), and an early use of DLI (from day +90) in case of MRD positivity, either by flow cytometry or by RT-qPCR on target genes at least in acute leukemias, should be explored in the context of multicentric prospective trials.

The other way to prevent relapse in acute leukemias is the use of post-transplant maintenance with molecular target drugs (e.g. FlT3 inhibitors, IDH inhibitors, BCL2 inhibitors,….) or de-methylating agents (e.g. oral azacitidine or decitabine) once again driven by MRD assessment. Antar et al. contribution was a very comprehensive review of these approaches, with a detailed algorithm for clinical use. Liu et al. report on a prospective use of low-dose decitabine as maintenance in patients with acute lymphoblastic leukemia (ALL), suggesting that this strategy could be promising in T-cell ALL. Overall, an early assessment of MRD (e.g. day +60/+90) may offer the opportunity to design a patient-specific protocol for relapse prevention, which should consider all the above-mentioned approaches. In particular, focusing on acute leukemias, the combination of immunotherapy (pre-emptive DLI) with molecular target therapies or de-methylating agents is intriguing and should be explored in multicentric trials (**Figure 1**).

**Figure 1:**
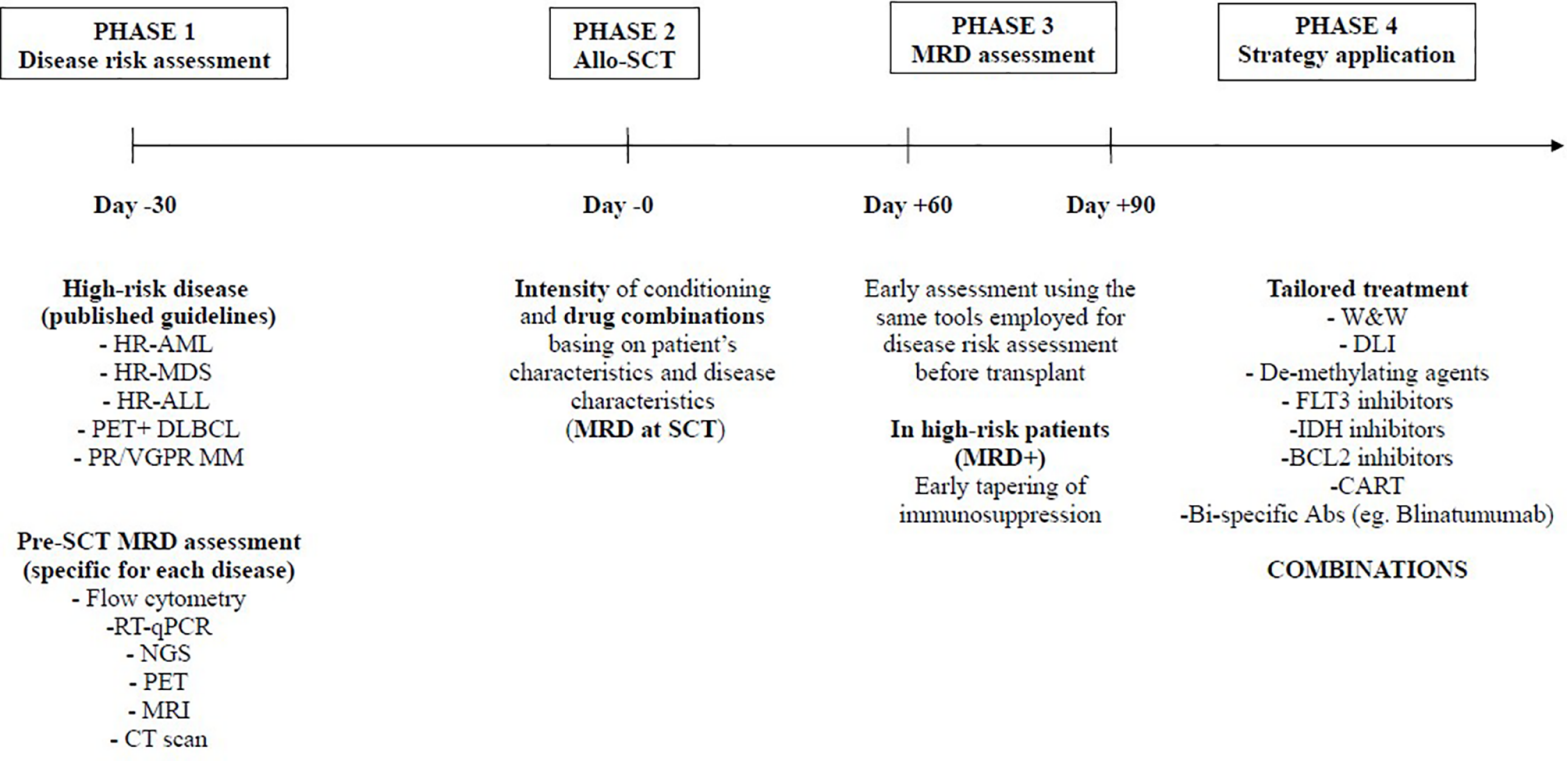
Relapse prevention following allo-SCT.

Many other manuscripts of this Research Topic cover topics related to transplant toxicity and non-relapse mortality, either focusing on conditioning intensity and stem cell source, or on CMV prophylaxis with letermovir, or on microbiome assessment and preservation, or on immune reconstitution monitoring.

Li et al. showed that decitabine could be used as conditioning regimen for myelodysplastic syndromes, allowing better long-term outcome with respect to decitabine alone without allo-SCT. Ma et al. confirmed previous observations that peripheral blood stem cells could be safely used in haploidentical transplantation, leading similar outcomes in comparison to a mix of bone marrow and peripheral blood stem cells. Finally, Song et al. reviewed the available data in the literature on the topic of conditioning intensity in acute myeloid leukemia and myelodysplastic syndromes, showing that reduced intensity conditioning regimens may be as effective as conventional regimens. Overall, the issue of conditioning intensity and drugs used in the conditioning is still a matter of debate, and in the last 15 years, we observed very different approaches, moving from very low intensity reduced intensity conditioning regimens, to reduced-toxicity regimens with new drug combinations (e.g. busulfan+fludarabine, treosulfan,…) (8, 9), to very intensive treatments (sequential conditioning) (10). This point clearly highlights that the choice of the conditioning needs to be patients-based, considering the type of disease, the co-morbidities, the frailty and the type of donor.

Terao et al. report on the effects of letermovir on T-cell reconstitution following haploidentical SCT with post-transplant cyclophosphamide and suggest that it may increase the levels of HLA-DR+ T-cells that may be implicated in the development of cGVHD. The use of letermovir for CMV prophylaxis between day 0 and +100 probably represents one of the most important advances in allo-SCT in the last 10 years. Both the registrative trial and several real-life analyses confirm that the incidence of CMV clinically significant infections dropped from more than 50% in the pre-letermovir era to 10-15% in the letermovir era (11, 12). Nevertheless, the issue of CMV in allotransplanted patients still need to be investigated, in particular concerning T-cell reconstitution during letermovir prophylaxis (is it delayed)? and the impact of late CMV reactivations after day +100, when letermovir is discontinued (the results of the multicentric randomized trial with letermovir from day +100 to day +200 are highly awaited). Allo-SCT platforms using post-transplant cyclophosphamide, which has been increasingly investigated in both haploidentical and non-haploidentical settings, appears to be associated with increased viral reactivation rate (13). While letermovir introduction might be a turning point in CMV reactivation prevention, other viruses are still lacking specific management (13). Moreover, deep relationships between intestinal microbiota composition and allo-SCT outcomes have been identified (14), particularly for predicting the mortality from infectious and non-infectious causes. Furthermore, therapeutic manipulations of the gut microbiota, such as fecal microbiota transplant, have emerged as promising therapeutic approaches for restoring the intestinal microbiota post-transplantation (15).

The topic of T-cell reconstitution and its interplay with microbiome has been covered in 3 articles. Milano et al. analysed the repertoire of TCR following cord-blood transplantation and showed that high TCR diversity at day +28 is associated with better patient outcomes. Andrlová et al. approached the topic of immune reconstitution focusing on unconventional T cells, specifically mucosal-associated invariant T cells, γδ-T cells, and invariant NK T cells. Evidences from published data suggest that the near future will face the development of pre-clinical and clinical trials exploring the manipulation of unconventional T-cell compartment and the interplay between microbiota and these unconventional cells. In line with this Research Topic, Alexander et al., on behalf of the Autoimmune Diseases Working Party of the EBMT, focused on the available data on microbiome perturbance following SCT for autoimmune diseases, showing that dysbiosis may influence the outcome in this setting of patients, as observed for conventional allo-SCT for hematological malignancies. Finally, Serpenti et al. showed that immune reconstitution may be useful for predicting severity of cGVHD and long-term outcome, by calculation of a risk-score at cGVHD onset. All these manuscripts clearly underline how intriguing is the topic of immune reconstitution and how complex are the connections with other biological aspects such as microbiome.

Finally, there is an urgent need to re-think the role of allo-SCT in certain diseases (e.g. aggressive lymphomas). This topic has been covered by a review published in the Research Topic by Goldsmith et al. At present, only relapsed and refractory diffuse large B cell lymphomas (DLBCL), primary mediastinic B cell lymphoma (PMBCL) and B-cell acute lymphoblastic leukemia (B-ALL) in patients younger than 25 years have a clear access to CART-cell therapies, in presence of non-responsive residual disease (16). Following the impressive results of the third line therapy with CART, the role of allo-SCT appears even more restricted to very high risk patients in complete remission. In this view, using the CAR-T therapy as a bridge to transplant may be an attractive but questionable option, considering the very high cost of CAR-T. Recently, EBMT and EHA proposed a revised version of recommendations to guide the delivery and management of CART-cell therapies (17). In the next future, CART will be available in many Countries for many other lymphoproliferative diseases such as mantle cell lymphoma, chronic lymphocytic leukemia and multiple myeloma and moreover for acute myeloid leukemias (AMLs). In this latter case, CART could be competitive with allo-SCT or complementary as treatment able to induce complete remission in refractory AML, before eradication with allo-SCT.

In summary, considering the articles published in this Research Topic, we can say that allo-SCT is going through a very enthusiastic phase of research and integration with novel strategies. Relapse prevention with maintenance or pre-emptive treatments, immune reconstitution and microbiome monitoring, modulation of conditioning intensity and integration with CART-cell therapy are some of the active Research Topics that the transplant community will deal with in the next future. Moreover, haploidentical transplantation, in particular followed by post-transplant cyclophosphamide GVHD prophylaxis, has been proved to be as effective as or even superior to HLA-matched allo-SCT. This clearly opened a new scenario, in which a very high risk disease, such as, for example, AML with MRD persistence, can be rapidly and successfully addressed to allo-SCT in first CR (18, 19). Allo-SCT will ultimately be a tailored therapy: not one transplant for many patients, but one transplant for one patient.

## Author Contributions

All the authors edited the Research Topic. MMa, DR, GR, and JP wrote the editorial. All authors contributed to the article and approved the submitted version.

## Conflict of Interest

The authors declare that the research was conducted in the absence of any commercial or financial relationships that could be construed as a potential conflict of interest.

## Publisher’s Note

All claims expressed in this article are solely those of the authors and do not necessarily represent those of their affiliated organizations, or those of the publisher, the editors and the reviewers. Any product that may be evaluated in this article, or claim that may be made by its manufacturer, is not guaranteed or endorsed by the publisher.
